# An Optimized Protocol for Packaging Pseudotyped Integrase Defective Lentivirus

**DOI:** 10.1186/s12575-016-0044-z

**Published:** 2016-07-11

**Authors:** Ranjita Sengupta, Chandreyee Mukherjee, Nandita Sarkar, Zhihong Sun, Jacob Lesnik, Joseph Huang, Biao Lu

**Affiliations:** System Biosciences (SBI), 265 North Whisman Rd., Mountain View, CA 94043 USA; Gilead Sciences, Inc., 333 Lakeside Dr., Foster City, CA USA; Department of Bioengineering, School of Engineering, Santa Clara University, 500 El Camino Real, Santa Clara, CA 95053 USA

**Keywords:** Integrase defective lentivirus, lentiviral package, viral transduction

## Abstract

**Background:**

A number of integrase defective lentiviral (IDLV) packaging systems have been developed to produce integration deficient lentiviruses for gene delivery and epichromosomal expression. However, despite their growing demand, a comparative study to systemically evaluate the performance efficiency of different mutants on virus packaging and gene expression has not been done.

**Results:**

Site-directed mutagenesis was used to generate five integrasedeficient mutants for non-integrative lentiviral packaging (NILVP). The five mutants were then individually incorporated to make different integrase defective lentivirus plasmid packaging mix, keeping other packaging factors constant. CD511B-1, a lentivectorexpressing GFP from an EF1 promoter, was packaged with each of the five different lentivirus packaging mix to make pseudotypedviral particles. The performance and packaging efficiency of each of the integrase deficient mutants was evaluated based on GFP expression in HT1080 cells, while the wild type lentivirus packaging mix was used as a control. Of the five integrase mutant candidates, one with the highestGFP transgene expression level was chosen for further characterization. The non-integrative nature of this candidate was confirmed by quantitative polymerase chain reaction and characterized using both dividing and non-dividing cells. Finally, a detailed standard protocol for NILVP using this integrase defective mutant was developed.

**Conclusions:**

An efficient lentiviral packaging system for producing on-integrative lentivirus was established. This system is compatible with most existing lentivectors and can be used to transduce both dividing and non-dividing cells.

**Electronic supplementary material:**

The online version of this article (doi:10.1186/s12575-016-0044-z) contains supplementary material, which is available to authorized users.

## Background

Lentiviral vectors provide one of the most effective gene delivery systems that have a broad range of applicationsfrombasic research to gene therapy [1–3]. For instance, HIV-basedlentivectorshave been extensively used for stably expressing different effector molecules, including cDNA, siRNA, long non-coding RNA, DNA fragment, antisense, ribozyme, and transcriptional reporter [4–6]. Recently, lentiviral vectors have been used clinically to express chimeric antigen receptor (CAR) in T lymph cells, enabling CAR-T cells in curing end-stage B cell leukemia [7, 8]. By packaging the lentiviral construct into pseudoviral particles, an efficient transduction can be achieved even with the most difficult cell types, such as primary cells, stem cells and hematopoietic cells [1, 2]. Lentivectorsalsohave a larger gene-cargocapacity (~7–8 kb) with low immunogenicity compared to other delivery vehicles. The major disadvantage of lentivectorsisits ability to integrate into the genome, which can lead to insertion mutations and other side effects [1, 9].

To circumvent this, one can generate non-integrative lentivirusesthat remain in the cell epichromosomally. This episomal vector can retain all the advantages of lentivectorsand express transgene of interest without causinginsertional mutations. There are several methods to generate non-integrative lentivirus and one such approach is to mutate the integrase gene. Although studies have identified several critical amino-acids for generating so called integrase defective lentivirus (IDLV) [10–12], a comparative study to systemically evaluate their relative performance on gene expressionislacking.

To make lentiviral particles, lentivectors which carry the transgene of interest (in this case GFP under a constitutive promoter EF1α, CD511B-1) is mixed with a plasmid packaging mix which includes the integrase, envelope gene and other genes necessary to generate pseudoviralparticles. This plasmid mix is then co-transfected into a 293 T producer cell line. Viral harvest is done 48 h and 72 h post transfection. In this study, we designed and generated a cohort of integrase mutants by site directed mutagenesis. We then evaluated the packaging efficiency of each of the mutants when combined with rest of the lentivirus packaging plasmids and identified one with the highest packaging efficiency. We further evaluated and characterized this mutant for its ability totransduceboth dividing and non-dividing cells. Since the mutation occurs in one of the packaging plasmids, this system is likely compatible with most existing lentivectors, hence providing an efficient and robust system for IDLV production.

## Results and Discussion

### Lentiviral Packaging System and IDLV Production

**Fig. 1:**
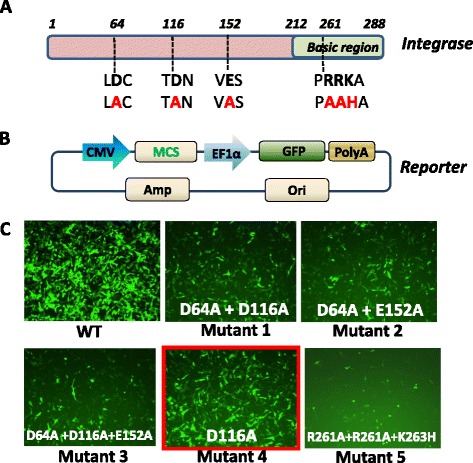
System design and screening of IDLV. Schematic representation of the HIV-1 integrase domain and its basic region (**a**). Numbers correspond to amino acid position of integrease domain. Site-directed mutagenesis resulted in mutation of the wild-type amino acids (bold black letters) to mutants (bold red letters). An illustration of a lenti-reporter used to assess the transduction and expression of each mutant. The reporter has dual promoter, CMV and EF1α, MCS are multiple cloning sites for potential insertion of gene of interest. PolyA isthe signaling sequences of polyadenylation (**b**). HT1080 cells were imaged after 72 h transduction and the best performer in mutant group was highlighted in red (**c**)

Currently, the most efficient technology for producing high titer, replication-incompetent, and infectious lentiviral particles, is based on transient and coordinated expression of a lentiviral vector along with plasmids expressing all the necessary packaging proteins delivered into producer cells by simultaneous transfection [13]. When expressed in packaging cells, the lentivector produces large number of the transcriptcontaining all functional elementsrequired for efficient packaging, including cPPT, Psi and RRE [13]. In order to be compatible with existing lentiviral vectors, we chose to modify one of the packaging proteins, polymerase (pol) for IDLV production. The polymerase contains both the polymerase domain and the integrase domain for efficient integration of transgene into the genome. Specifically, we mutated three critical amino-acids and one triplet of integrase within the pol gene. A schematic diagram is shown in Fig. 1a, where the location of the mutations in the Integrase domain is shown. These mutations will produce defective integrase during lentiviral packaging. The integrase defective Pol plasmid can now be used to produce integrase defective lentiviral particles, without requiring any further modifications to the lentivector backbone.

We initially generated 5 different mutants in the integrase gene (Fig. 1a) and selected the best performer with respect to gene expression. To achieve this, we used a previously established green fluorescent reporter expressed under EF1α promoter CD511B-1 as a read-out (Fig. 1b). We usedWT Integrase and candidate Integrase mutants along with Gag and VSVG packaging plasmid mix to package CD511 under identical experimental conditions. As shown in Fig. 1c, all mutant candidates express GFP at varying levels, albeit much lower than the GFP expressionfromthe WT control, whichissimilar to published reports [10–12]. Among them, mutant D116Ademonstrated the highest levels of reporter expression (Fig. 1c). Since we used the same amount of infectious viral particles to perform the transduction, we selected mutant D116A for further characterization.

**Fig. 2:**
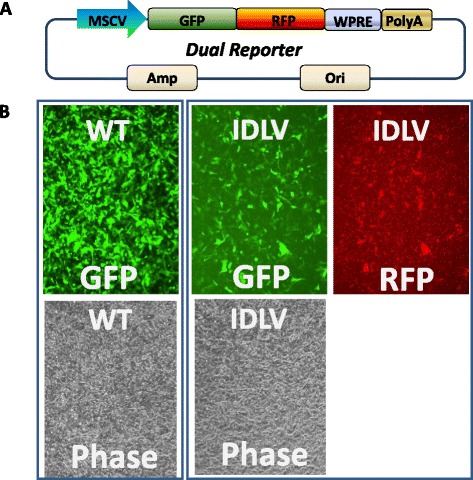
A comparative study of WT and IDLV. **a** lentivector expressing GFP and RFP from MSCV promoter (LV605A-1) was packaged with wild-type and mutant integrase; WPRE are woodchuck hepatitis virus-derived posttranscriptional regulatory element for stabilizing the transcripts. The same amount of packaged psudoviruses was used to transduce HT1080 cells. **b** Images were taken at Day 3 after transduction

To test if mutant D116A could express other transgenes, we used a different lentivector reporter combination, a mouse stem cell viral promoter driven dual reporter construct MSCV-GFP-T2A-RFP (LV605VA-1), Following viral transduction, the MSCV-driven GFP-RFP fusion vector expressed GFP and RFP, suggesting that D116A mutant is able to produce IDLV viruses for transgene expression using a different promoter and reporter (Fig. 2). Based on the results above, we were able to identify and confirm the best mutant among these five integrase mutants. This mutant (D116A) can be used to package IDLV for gene transduction and expression in mammalian cells. The information concerning the expression vector of mutant D116A and its encoding sequences is provided in Additional file 1: Figure S1.

### D116A Mutant Produces IDLV with Non-Integration Nature

**Fig. 3:**
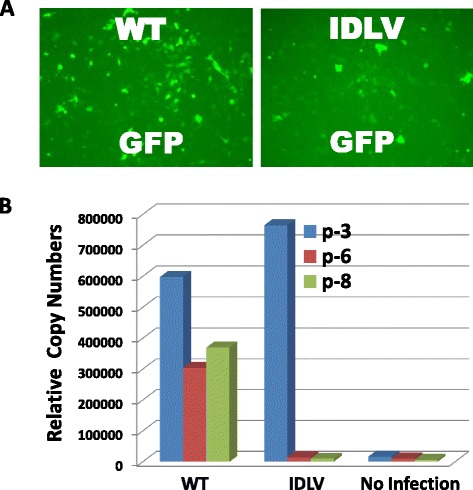
Non-integrative nature of IDLV. **a** Expression of GFP reporter after lentiviral transduction on Day 3. **b** A real-time PCR analysis of relative viral copy numbers at different passages in HT1080 cells. P-3, p-6, and p-8 are cell passage numbers counted post-transduction with WT and IDLV reporter viruses

To rule outthe possibility that the D116A mutant may produce integrative lentiviral particles rather than non-integrative lentivirus, we next carried out a comparative transduction experiment on HT1080 cells. After transducingcultured cells with GFP reporter virus CD511VB-1, both the wild-type and integrase defective viral particles showed GFPexpression at Day 3 (Fig. 3a). If D116Amutant produced true non-integrative IDLVparticles, the copy numbers of the transgene would decrease with every cell division and passage, since transgenes are episomal and cannot replicate along with the genomic DNA. Alternatively, if they do integrate into the genomic DNA, they will be able to replicate along with cell growth and division. Therefore, we conducted a quantitative real-time PCR analysis to examine the relative copy numbers of transgenes. While the WT integrase virusshowed an initial decrease in copy number at passage 6, which was relatively stable until passage 9, D116A mutant demonstrated a steady decrease in copy numbers that were close to levels of the non-infected control (Fig. 3b). Thus, D116A mutant produces non-replicable and non-integrative lentiviral particles, which is consistent with results from previous reports using either PCR or Southern blot analysis [10–12].

### IDLV Tranducesboth Dividing and Non-Dividing Cells

**Fig. 4:**
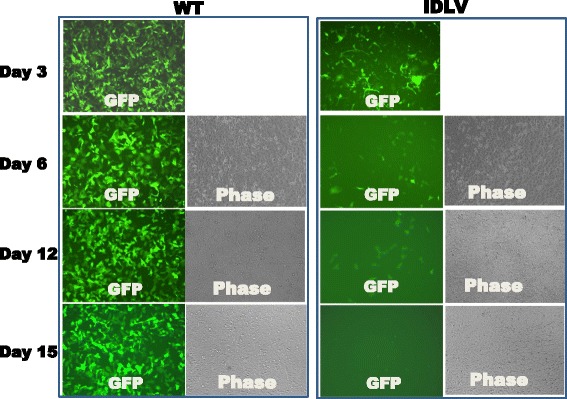
Transgene expression in dividing cells. HT1080 cells were imaged at different days after transduction. Images were taken under the same exposure setting to compare the relative expression levels of the GFP reporter when wild-type integrase or D116A mutant were used

We next examined the performance of IDLV for transducing dividing and non-dividing cells. To study transgene expression in dividing cells, we transduced HT1080 with GFP reporter virus (CD511VB-1), and compared the relative expression levels between wild-type and mutant viruses by monitoring their GFP expression. As shown in Fig. 4, HT1080 cells transduced with WT CD511 virus produced high levels of GFP expression through 3 passages, suggesting permanent expression due to transgene integration. In contrast, HT1080 cells transduced with the integrase defective mutant CD511 virus resulted in an initial expression of GFP, followed by a steady decrease in both the intensity of GFP expression levels and percentage of GFP positive cells (Fig. 4 right panel). These results were consistent with previous reports [10–12], indicating the transient expression nature of IDLV when used to transduce dividing cells.

**Fig. 5:**
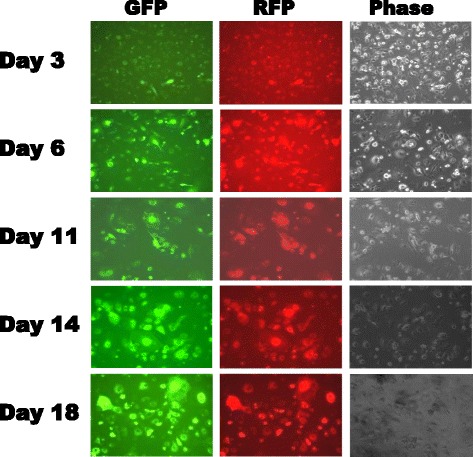
Transgene expression in nondividing cells. Mouse embryonic fibroblasts (STO) were treated with actinomycin to make nondividing cells. The treated cells were subsequently transduced with IDLV reporter viruses. Images were taken using either GFP-filter (left panel), RFP-filter (middle panel) or phase contrast (right panel) to show the reporter expression

We next assessed the performance of IDLV transduction in non-dividing cells where the transgene should remain stable. We first prepared non-dividing cells by treating the mouse embryonic fibroblast cell (STO) with actinomycin [14]. Subsequently we performed transduction with theMSCV-GFP-RFP dual reporter virus, resulting in expression of both GFP and RFP in these non-dividing STO cells. The GFP and RFP expression were persistent and even increased slightly up to Day 18 after transduction (Fig. 5), showing that transgene expression is relatively stable in those non-dividing cells. Our results are consistent with a previous study that IDLV may have a long-term and sustainable transgene expression in non-dividing cells. Hence these features of IDLV may be exploited when non-proliferative tissues are targeted.

Lentivirus has been used transducing some primary and hoard-to-transfect cells. Although we did not provide direct evidence in this study, we expect IDLV will follow the tropism of wild-type counterpart, because this change occurs within the integrase domain of POL gene, which will not directly affect the tropism of lentivirus.

### IDLV Performance in Relationship with Its Cargo Size

**Fig. 6:**
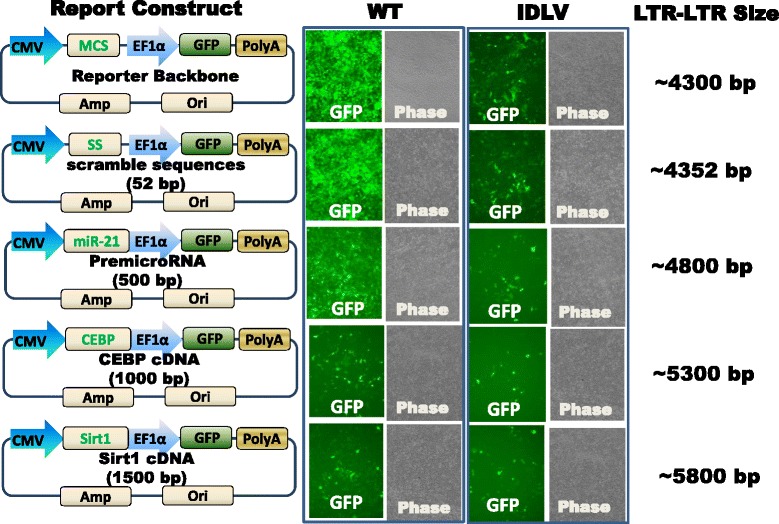
Gene expression levels decrease with incremental cargo size for both wild-type and IDLV. Viral particles were packaged with both wild-type and mutant integrase. The same amounts of viral particles were used for transducing HT1080 cells. Images were taken at Day 3 after transduction. LTR are long terminal repeats derived from HIV provirus

We next determined whether gene size also influences the gene expression level. We first generated a series of lentivectors with increasing cargo size. To monitor gene expression, we used the constitutive promoter EF1α to drive the GFP reporter, and another gene expression cassette to accommodate various sized genes under the control of a CMV promoter. With the incorporation of gene fragments increasing incrementally in size (Fig. 6), we observed a steady decrease in the levels of reporter expression. This decrease occurred in both WT and IDLV groups, suggesting the cargo size is an important factor when using a lentivector.

## Conclusion

We have identified and validated an integrase mutant that can be used to produce non-integrative lentiviral particles. This IDLV system would allow researchers to use their existing lentivectors to generate non-integrative lentiviral particles with increased safety profile and decreased side effect such as insertional mutation and insertion-caused genome instability respectively.

## Methods

### Integrase Mutagenesis and Reporter Construction

The pPACKH1-GAG plasmid (SBI, Mountain View, CA) contains the structural (gag), replication (pol) and envelop (env) gene. The pol contains a functional domain of integrase, which is responsible for provirus integration. As shown in Fig. 1a, we designed and synthesized primer pairs to carry out site-directed mutagenesis using high fidelity DNA polymerase followed by DpnI (NEB, Ipswich, MA) treatment and transformation. The primers used for D64A (forward 5′- aggaatatggcaactagcttgtacacatttagaag-3′; reverse 5′-ctaaatgtgtacaagctagttgccatattcctggac), D116A (forward 5′- gtaaaaacaatacatacagccaatggcagcaatttcacc-3′; reverse 5′- gaaattgctgccattggctgtatgtattgtttttactggc-3′), D152A (forward 5′-caaagtcaaggagtagtagcatctatgaataaagaattaaagaaaatt; reverse 5′- ctttaattctttattcatagatgctactactccttgactttgggg-3′), and triplet mutation in basic region (forward5′-gtgacataaaagtagtgccagcagcacatgcaaagatcattagggat-3′; reverse primer 5′-atccctaatgatctttgcatgtgctgctggcactacttttatgtcac- 3′). Using different combinations of primer pairs, we have generated five integrase mutants as confirmed by double stranded DNA sequencing (SeqeneTech, Mountain View, California).

### Cell Culture and Transduction

Human embryonic kidney HEK293-TN (LV900A-1, SBI, Mountain View, CA) and fibrosarcoma HT1080 (ATCC, Manassas, VA) cells were maintained in high glucose Dulbecco’s Minimal Essential Medium (DMEM) supplemented with 10 % FBS, 2 mMGlutaMax (Life Technologies), 100 U/ml penicillin and 100 U/ml streptomycin. Transduction reagent TransDux (LV850A-1, SBI, Mountain View, CA) was used to infect cells with virus to enhance the transducing efficiency. CD511B-1 (GFP reporter) and LV605A-1 (DualGFP-RFP reporter) from SBI were the constructs used to make WT and integrase defective lentiviral particles for most of the studies. These constructs were assembled by a combination of PCR and seamless fusion method as reported before [15, 16].

### STO Culture and Actinomycin Treatment

STO cells were cultured in DMEM high-glucose (Life Technologies), supplemented with 2 mMGlutaMax (Life Technologies), and 1 % nonessential amino acid (Life Technologies). Cells were treated with 10 μg/ml actinomycin C (Sigma) for 3 h. The actinomycin C-treated STO non-dividing cells were extensively washed in PBS and plated at 35, 000 cells/cm^2^ in gelatin-coated tissue culture dish.

### Microscopy and Reporter Gene Assay

All microscopy was performed on live cells with a LEICA DMI3000B microscope following 48 ~ 72 h transduction. Data collection and processing were performed with LAS 3.8 software.

### Lentiviral Titration

Pseudoviral titer was performed using a real time PCR based kit (LV961A-1, SBI, Mountain View, CA). This kit measures the copy numbers of integrated gene after transduction. Briefly, HT1080 cells were transduced in the presence of 1 × TransDux reagent (LV850A-1, SBI, Mountain View, CA) for 72 h. Subsequently cells were lysed and DNAs were isolated according to manufacturer’s instructions. PCR reactions were performed with primers for amplifying a conserved genomic DNA sequences or WPRE in the transgeneusing Applied Biosystems 7300 Real time PCR System. PCR condition: 50 °C, 2 min; 95 °C, 10 min; followed by 40 cycles of 95 °C, 15 s and 60 °C for 1 min, followed by a dissociation step.

### Standard Operating Procedure for Lentiviral Packaging

#### Reagents

The expression constructs CD511B-1 and LV605A-1 (SBI, Mountain View, CA) are packaged into VSVG pseudotypedviral particles with proteins produced by the lentiviral packaging plasmid mix (LV500A-1, SBI, Mountain view, CA). The lentiviralpackaging system consists of an optimized mixture of three plasmids, including pPACKH1-GAG, pPACKH1-REV and pVSVG. To produce integrative competent lentiviruse, the pPACKH1-GAG has a wild-type pol gene. To produce non-integrative lentiviruses, the pPACKH1-GAG with a mutated integrase (an integrase domain in pol gene) was used instead of the wild-type. Other reagents include virus producer cell lineHEK293TN (LV900A-1, SBI, Mountain View), transfection reagent PureFection (LV750A-1, SBI, Mountain View), and virus precipitation solution (LV810A-1, SBI, Mountain View).

Additional reagents include DMEM high glucose with sodium pyruvate and l-glutamate (Invitrogen, Cat. #111995073), Fetal bovine serum (Invitrogen, Cat. #16000036), Penicillin/Streptomycin (Invitrogen, Cat. # 15070063), Trypsin-EDTA (Sigma, Cat. #T3924), hematopoietic plate cell counter.

### Step-by-step Protocol (bench time 110 min, total time 6 days)

#### Day 1

Seed 293 TN producer cells (30 min)

18 ~ 24 h prior to transfection, wash cells with pre-warmed DPBS once and add 2 mlTrypsin-EDTA to the culture flask. Incubate for 5 min or until cells become detached from surface and add 10 ml of complete medium to neutralize the digestion enzyme. Precipitate cells by centrifugation at 300 rpm for 5 min at room temperature. Re-suspend cell pellet in 5 ml complete medium and count cells. Seed 293TN cells at 7 ~ 8 × 10^6^/15 cm^2^ in 20 ml complete culture medium with antibiotics, until the cell density reaches 70 ~ 80 % confluence for transfection.

#### Day 2

Transfecting 293TN Producer cells with lentivector expressing reporter (CD511, LV605) and lenti viral packaging plasmid mix expressing WT and mutant integrase (bench time 30 min)

1. *Prepare lentivector DNA and packaging plasmid mix*
  Add 1 ~ 1.6 ml plain DMEM (serum and antibiotic free) to an Eppendorf tube; add 4.5 μg lentivector and 45 μl packaging plasmid DNAinto the DMEM. Mix by pipetting for 10 times.
2. *Prepare transfection mix*
  Add 55 μl PureFection into the same tube. Vertex for 10 s and incubate the DMEM-Plasmid-PureFectionmixture at room temperature for 15 min.
3. *Apply transfection mix to producer cells*
  Add DMEM-Plasmid-PureFectionmixture drop-wise to the dish, and swirl to disperse evenly; return the dish to the cell culture incubator at 37 °C with 5 % CO_2_; change the medium 12 ~ 24 h after transfection (optional).

#### Day 4 and Day 5

Harvest packaged viruses (bench time 30 min; total 2 h)

48 h after transfection, collect the culture medium containing pseudoviral particles into a 50-ml sterile, capped conical centrifuge tube and store at 4 °C until next harvest. Re-fill producer cells with 10 ml fresh complete medium slowly, and return dishes to the cell culture incubator.

72 h after transfection, collect the culture medium and combined it with the collection at 48 h. Centrifuge at 3000 × g for 15 min at room temperature to pellet cell debris. Transfer the viral supernatant into a new tube and add 1 volume of cold PEG-it Virus Precipitation Solution (4 °C) to every 4 volumes of Lentivector-containing supernatant. Larger volume precipitation of lentiviral particles can be achieved by using the Corning 250 ml polypropylene centrifuge tube (Cat. #430776), following manufacturer’s instructions: refrigerate overnight or for at least 12 h. Lentivirus-containing supernatants mixed with PEG-it Virus Precipitate Solution (LV810A-1, SBI, Mountain View, CA) are stable for up to 4 ~ 5 days at 4 °C.

#### Day 6

Collect and concentrate psuedotyped viral particles (bench time 20 min; total 1.2 h)

Centrifuge supernatant/PEG-it mixture at 3000 × g for 30 min at 4°C, and transfer supernatant to a fresh tub. After centrifugation, the lentivector particles will appear as a beige or white pellet at the bottom of the vessel. Spin down residual PEG-it solution by centrifugation at 3000 × g for 5 min; remove all traces of fluid by aspiration, and avoid disturbing the precipitated lentiviral particles in pellet. Re-suspend lentiviral pellets from 1/10 to 1/100 of original volume using cold, sterile PBS or DMEM containing 25 mM HEPES buffer at 4 °C. Aliquot into cryogenic vials and store at −70 °C.

### Trouble-Shooting Advice

1. Low viral titer (<105 ifu/mL) can be caused by a number of reasons. The most common ones include:(1) poor transfection efficiency due to too high or too low density of producer cells. (2) Poor quality of lentivector or packaging plasmid DNA. (3) Inadequate ratios of plasmid mix to the transfection reagent. To avoid these, plate cells to achieve 70 ~ 80 % confluency at transfection stage and use endotoxin-free plasmid purification kit to prepare lentivector expressing the transgene and packaging plasmids.
2. If problem remains, other factors to be consideredare: (1) producer HEK293TN cells are of poor quality due to over confluence; (2) cells may be contaminated by mycoplasma; (3) too many passages (>20) of cells. In those cases, one should revive a new batch of producer cells and maintain them in the logarithmic growth phase.
3. Other factors which could affect virus quality are: The packaging limit for the lentiviral system, which is ~8.5 kb from 5′ → 3′ LTR. The efficiency of packaging drops significantly when cDNA size increases. For a 3 kb insert, the titers and expression level can be 10-fold less compared to that ofa 1 kb insert. Repeated sequences can also cause problems in transgene expression as recently reported [17].

### Safety Guide to Packaging and Transduction of Target Cells

The use of HIV-based pseudotypedlentiviruses falls within NIH Biosafety Level 2 criteria due to the potential biohazard risk of possible recombination with endogenous viral sequences to form self-replicating virus, or the possibility of insertional mutagenesis. For a description of laboratory biosafety level criteria, consult the Centers for Disease Control Office of Health and Safety Web site. It is also important to check with the health and safety guideline at your institution regarding the use of lentiviruses and always follow standard microbiological practices.

## Abbreviations

CMV, cytomegalovirus promoter; EF1-α, elongation factor 1- alpha promoter; GFP, green fluorescent protein; IDLV, integrase defective lentivirus; MSCV, murine stem cell virus promoter; NILVP, non-integrative lentiviral packaging; RFP, red fluorescent protein; VSVG, vesicular stomatitis virus envelope glycoprotein

## Additional file

Additional file 1: Figure S1. Schematic representation of the packaging plasmid, expressing GAG, POL, TAT and ENV. Panel A shows the configuration of the expression vector for wild-type pol gene, whereas panel B shows the site-directed mutant, resulting in the change of D116A (denoted by the red star). (PDF 590 kb)
