# Supplementary material for: An Optimized Protocol for Packaging Pseudotyped Integrase Defective Lentivirus
Source: Biol Proced Online. 2016 Jul 11;18:14. doi: 10.1186/s12575-016-0044-z (PMC4939624; doi:10.1186/s12575-016-0044-z)
Supplement: Additional file 1: Figure S1. — Schematic representation of the packaging plasmid, expressing GAG, POL, TAT and ENV. Panel A shows the configuration of the expression vector for wild-type pol gene, whereas panel B shows the site-directed mutant, resulting in the change of D116A (denoted by the red star). (PDF 590 kb) [file 12575_2016_44_MOESM1_ESM.pdf]

Additional file 1: Figure S1

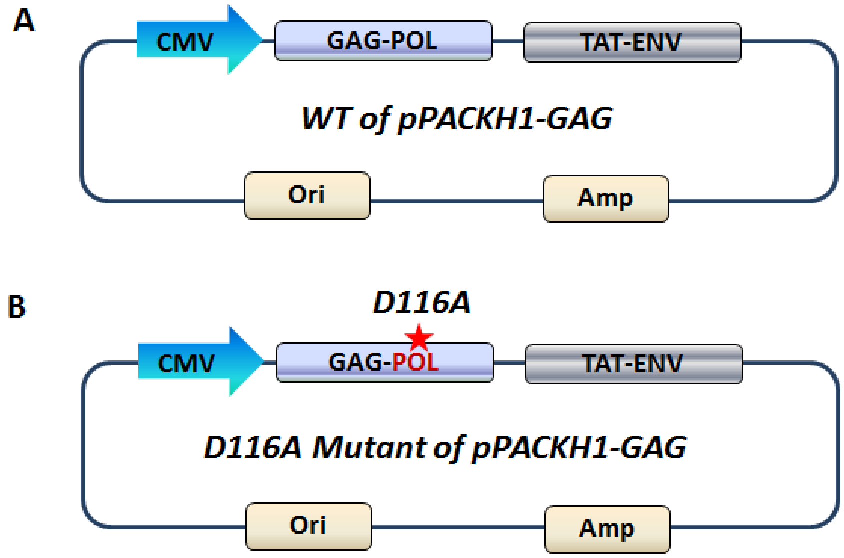

Figure S1. Schematic representation of the packaging plasmid, expressing GAG, POL, TAT and ENV. Panel A shows the configuration of the expression vector for wild-type pol gene, whereas panel B shows the site-directed mutant, resulting in the change of D116A (denoted by the red star).

>Wild-type GAG and POL coding sequence

atgagtttgccaggaagatggaaaccaaaaatgatagggggaattggaggttttatcaaagtaagacagtatgatcagatactcatagaaatctgtggacataaagctata
ggtacagtattagtaggacctacacctgtcaacataattggaagaaatctgttgactcagattggttgcactttaaattttcccattagccctattgagactgtaccagtaaaatt
aaagccaggaatggatggcccaaaagttaaacaatggccattgacagaagaaaaaataaaagcattagtagaaatttgtacagagatggaaaaggaagggaaaatttca
aaaattgggcctgaaaatccatacaatactccagtatttgccataaagaaaaaagacagtactaaatggagaaaattagtagatttcagagaacttaataagagaactcaag
acttctgggaagttcaattaggaataccacatcccgcagggttaaaaaagaaaaaatcagtaacagtactggatgtgggtgatgcatatttttcagttcccttagatgaagac
ttcaggaaatatactgcatttaccatacctagtataaacaatgagacaccagggattagatatcagtacaatgtgcttccacagggatggaaaggatcaccagcaatattcc
aaagtagcatgacaaaaatcttagagccttttagaaaacaaaatccagacatagttatctatcaatacatggatgatttgtatgtaggatctgacttagaaatagggcagcata
gaacaaaaatagaggagctgagacaacatctgttgaggtggggacttaccacaccagacaaaaaacatcagaaagaacctccattcctttggatgggttatgaactccat
cctgataaatggacagtacagcctatagtgctgccagaaaaagacagctggactgtcaatgacatacagaagttagtggggaaattgaattgggcaagtcagatttaccc
agggattaaagtaaggcaattatgtaaactccttagaggaaccaaagcactaacagaagtaataccactaacagaagaagcagagctagaactggcagaaaacagaga
gattctaaaagaaccagtacatggagtgtattatgacccatcaaaagacttaatagcagaaatacagaagcaggggcaaggccaatggacatatcaaatttatcaagagc
catttaaaaatctgaaaacaggaaaatatgcaagaatgaggggtgcccacactaatgatgtaaaacaattaacagaggcagtgcaaaaaataaccacagaaagcatagt
aatatggggaaagactcctaaatttaaactgcccatacaaaaggaaacatgggaaacatggtggacagagtattggcaagccacctggattcctgagtgggagtttgttaa
tacccctcctttagtgaaattatggtaccagttagagaaagaacccatagtaggagcagaaaccttctatgtagatggggcagctaacagggagactaaattaggaaaagc
aggatatgttactaatagaggaagacaaaaagttgtcaccctaactgacacaacaaatcagaagactgagttacaagcaatttatctagctttgcaggattcgggattagaa
gtaaacatagtaacagactcacaatatgcattaggaatcattcaagcacaaccagatcaaagtgaatcagagttagtcaatcaaataatagagcagttaataaaaaaggaa
aaggtctatctggcatgggtaccagcacacaaaggaattggaggaaatgaacaagtagataaattagtcagtgctggaatcaggaaagtactatttttagatggaatagat
aaggcccaagatgaacatgagaaatatcacagtaattggagagcaatggctagtgattttaacctgccacctgtagtagcaaaagaaatagtagccagctgtgataaatgt
cagctaaaaggagaagccatgcatggacaagtagactgtagtccaggaatatggcaactagattgtacacatttagaaggaaaagttatcctggtagcagttcatgtagcc
agtggatatatagaagcagaagttattccagcagaaacagggcaggaaacagcatattttcttttaaaattagcaggaagatggccagtaaaaacaatacatacagacaat
ggcagcaatttcaccagtgctacggttaaggccgcctgttggtgggcgggaatcaagcaggaatttggaattccctacaatccccaaagtcaaggagtagtagaatctat
gaataaagaattaaagaaaattataggacaggtaagagatcaggctgaacatcttaagacagcagtacaaatggcagtattcatccacaattttaaaagaaaaggggga
ttggggggtacagtgcaggggaaagaatagtagacataatagcaacagacatacaaactaaagaattacaaaaacaaattacaaaaattcaaaattttcgggtttattacag
ggacagcagaaatccactttggaaaggaccagcaaagctcctctggaaaggtgaaggggcagtagtaatacaagataatagtgacataaaagtagtgccaagaagaaa
agcaaagatcattagggattatggaaaacagatggcaggtgatgattgtgtggcaagtagacaggatgaggattag

>D116A Mutant of GAG and POL coding sequence

atgagtttgccaggaagatggaaaccaaaaatgatagggggaattggaggttttatcaaagtaagacagtatgatcagatactcatagaaatctgtggacataaagctata
ggtacagtattagtaggacctacacctgtcaacataattggaagaaatctgttgactcagattggttgcactttaaattttcccattagccctattgagactgtaccagtaaaatt
aaagccaggaatggatggcccaaaagttaaacaatggccattgacagaagaaaaaataaaagcattagtagaaatttgtacagagatggaaaaggaagggaaaatttca
aaaattgggcctgaaaatccatacaatactccagtatttgccataaagaaaaaagacagtactaaatggagaaaattagtagatttcagagaacttaataagagaactcaag
acttctgggaagttcaattaggaataccacatcccgcagggttaaaaaagaaaaaatcagtaacagtactggatgtgggtgatgcatatttttcagttcccttagatgaagac
ttcaggaaatatactgcatttaccatacctagtataaacaatgagacaccagggattagatatcagtacaatgtgcttccacagggatggaaaggatcaccagcaatattcc
aaagtagcatgacaaaaatcttagagccttttagaaaacaaaatccagacatagttatctatcaatacatggatgatttgtatgtaggatctgacttagaaatagggcagcata
gaacaaaaatagaggagctgagacaacatctgttgaggtggggacttaccacaccagacaaaaaacatcagaaagaacctccattcctttggatgggttatgaactccat
cctgataaatggacagtacagcctatagtgctgccagaaaaagacagctggactgtcaatgacatacagaagttagtggggaaattgaattgggcaagtcagatttaccc
agggattaaagtaaggcaattatgtaaactccttagaggaaccaaagcactaacagaagtaataccactaacagaagaagcagagctagaactggcagaaaacagaga
gattctaaaagaaccagtacatggagtgtattatgacccatcaaaagacttaatagcagaaatacagaagcaggggcaaggccaatggacatatcaaatttatcaagagc
catttaaaaatctgaaaacaggaaaatatgcaagaatgaggggtgcccacactaatgatgtaaaacaattaacagaggcagtgcaaaaaataaccacagaaagcatagt
aatatggggaaagactcctaaatttaaactgcccatacaaaaggaaacatgggaaacatggtggacagagtattggcaagccacctggattcctgagtgggagtttgttaa
tacccctcctttagtgaaattatggtaccagttagagaaagaacccatagtaggagcagaaaccttctatgtagatggggcagctaacagggagactaaattaggaaaagc
aggatatgttactaatagaggaagacaaaaagttgtcaccctaactgacacaacaaatcagaagactgagttacaagcaatttatctagctttgcaggattcgggattagaa
gtaaacatagtaacagactcacaatatgcattaggaatcattcaagcacaaccagatcaaagtgaatcagagttagtcaatcaaataatagagcagttaataaaaaaggaa
aaggtctatctggcatgggtaccagcacacaaaggaattggaggaaatgaacaagtagataaattagtcagtgctggaatcaggaaagtactatttttagatggaatagat
aaggcccaagatgaacatgagaaatatcacagtaattggagagcaatggctagtgattttaacctgccacctgtagtagcaaaagaaatagtagccagctgtgataaatgt
cagctaaaaggagaagccatgcatggacaagtagactgtagtccaggaatatggcaactagattgtacacatttagaaggaaaagttatcctggtagcagttcatgtagcc
agtggatatatagaagcagaagttattccagcagaaacagggcaggaaacagcatattttcttttaaaattagcaggaagatggccagtaaaaacaatacatacagCcaat
ggcagcaatttcaccagtgctacggttaaggccgcctgttggtgggcgggaatcaagcaggaatttggaattccctacaatccccaaagtcaaggagtagtagaatctat
gaataaagaattaaagaaaattataggacaggtaagagatcaggctgaacatcttaagacagcagtacaaatggcagtattcatccacaattttaaaagaaaaggggga
ttggggggtacagtgcaggggaaagaatagtagacataatagcaacagacatacaaactaaagaattacaaaaacaaattacaaaaattcaaaattttcgggtttattacag
ggacagcagaaatccactttggaaaggaccagcaaagctcctctggaaaggtgaaggggcagtagtaatacaagataatagtgacataaaagtagtgccaagaagaaa
agcaaagatcattagggattatggaaaacagatggcaggtgatgattgtgtggcaagtagacaggatgaggattag

Note: The underlined wild-type sequences of “acagacaat” encode “TDN”, where the D116A mutant sequences of “acagccaat” encode “TAN”, resulting a wild-type D changed into A at the position of 116 of the integrase domain within pol gene.
